# Super-resolution deep learning reconstruction for improved quality of myocardial CT late enhancement

**DOI:** 10.1007/s11604-025-01760-2

**Published:** 2025-03-12

**Authors:** Masafumi Takafuji, Kakuya Kitagawa, Sachio Mizutani, Akane Hamaguchi, Ryosuke Kisou, Kenji Sasaki, Yuto Funaki, Kotaro Iio, Kazuhide Ichikawa, Daisuke Izumi, Shiko Okabe, Motonori Nagata, Hajime Sakuma

**Affiliations:** 1 Department of Radiology, Mie University Graduate School of Medicine, 2-174 Edobashi, Tsu, Mie 514-8507 Japan; 2 Department of Radiology, Matsusaka Municipal Hospital, Matsusaka, Japan; 3 Department of Cardiology, Matsusaka Municipal Hospital, Matsusaka, Japan

**Keywords:** Cardiac imaging technique, Computed tomography angiography, Myocardial infarction, Deep learning, Image reconstruction

## Abstract

**Purpose:**

Myocardial computed tomography (CT) late enhancement (LE) allows assessment of myocardial scarring. Super-resolution deep learning image reconstruction (SR-DLR) trained on data acquired from ultra-high-resolution CT may improve image quality for CT-LE. Therefore, this study investigated image noise and image quality with SR-DLR compared with conventional DLR (C-DLR) and hybrid iterative reconstruction (hybrid IR).

**Methods and methods:**

We retrospectively analyzed 30 patients who underwent CT-LE using 320-row CT. The CT protocol comprised stress dynamic CT perfusion, coronary CT angiography, and CT-LE. CT-LE images were reconstructed using three different algorithms: SR-DLR, C-DLR, and hybrid IR. Image noise, signal-to-noise ratio (SNR), contrast-to-noise ratio (CNR), and qualitative image quality scores are in terms of noise reduction, sharpness, visibility of scar and myocardial boarder, and overall image quality. Inter-observer differences in myocardial scar sizing in CT-LE by the three algorithms were also compared.

**Results:**

SR-DLR significantly decreased image noise by 35% compared to C-DLR (median 6.2 HU, interquartile range [IQR] 5.6–7.2 HU vs 9.6 HU, IQR 8.4–10.7 HU; *p* < 0.001) and by 37% compared to hybrid IR (9.8 HU, IQR 8.5–12.0 HU; *p* < 0.001). SNR and CNR of CT-LE reconstructed using SR-DLR were significantly higher than with C-DLR (both *p* < 0.001) and hybrid IR (both *p* < 0.05). All qualitative image quality scores were higher with SR-DLR than those with C-DLR and hybrid IR (all *p* < 0.001). The inter-observer differences in scar sizing were reduced with SR-DLR and C-DLR compared with hybrid IR (both *p* = 0.02).

**Conclusion:**

SR-DLR reduces image noise and improves image quality of myocardial CT-LE compared with C-DLR and hybrid IR techniques and improves inter-observer reproducibility of scar sizing compared to hybrid IR. The SR-DLR approach has the potential to improve the assessment of myocardial scar by CT late enhancement.

## Introduction

Late gadolinium enhancement (LGE) magnetic resonance imaging (MRI) is widely utilized as a non-invasive benchmark for myocardial infarction and focal fibrosis [1]. Myocardial computed tomography (CT) late enhancement (LE) performed after coronary CT angiography (CCTA) allows assessment of myocardial scarring, given the similar pharmacokinetics of iodinate contrast agents in CT and gadolinium contrast agents in LGE [2, 3]. Various studies have shown substantial agreement in late enhancement between CT-LE and LGE-MRI [4, 5]. Nonetheless, the contrast between remote and hyper-enhanced myocardium is lower in CT-LE than in LGE-MRI [2, 3]. Employing low tube potential imaging is effective for CT-LE, allowing a reduction in the radiation dose and increased contrast enhancement. However, this technique incurs the disadvantage of increased image noise [6, 7].

Recent improvements in CT technology, including new image acquisition and reconstruction algorithms, have reduced image noise. In CT-LE, iterative reconstruction methods have successfully reduced image noise [8, 9]. Deep learning image reconstruction (DLR) is a recent approach for CT reconstruction that incorporates deep convolutional neural networks (DCNN) into the image reconstruction algorithm. The conventional DLR (C-DLR) algorithm integrates a DCNN trained to discern noise from signals using model-based iterative reconstruction datasets, yielding reduced image noise in CCTA and dynamic CT perfusion (CTP) compared to hybrid iterative reconstruction (hybrid IR) images [10]. Recently, super-resolution DLR (SR-DLR) algorithms have emerged, trained on true ultra-high-resolution CT (UHR-CT) images, to provide denoised images with heightened spatial resolution [11]. SR-DLR can be applied to 320-row CT to yield cardiac CT images with improved spatial resolution and reduced image noise during conventional volumetric whole-heart CT examinations. A recent study demonstrated that compared to C-DLR, SR-DLR reduces image noise and enhances image quality for CCTA [12]. However, the efficacy of these DLR algorithms in improving image noise and image quality for CT-LE is yet to be evaluated.

This study investigated image noise and quality of CT-LE reconstructed by SR-DLR as compared to C-DLR and hybrid IR.

## Materials and methods

### Study population

This retrospective analysis investigated a prospectively registered cohort from a single center (reference no. 210604-5-2). This study was approved by the institutional review board, and written informed consent for participation in the study had been obtained from each patient prior to enrollment. We retrospectively identified 30 consecutive patients (11 women; median age, 73 years, interquartile range [IQR] 61–77 years) with known or suspected coronary artery disease who underwent CCTA between April and September 2022. Patients with known allergies to iodinated contrast agent or contraindications for adenosine were not included in this study cohort. Heart rate (HR), electrocardiogram (ECG), and blood pressure were monitored during examinations. To assess radiation exposure, the dose-length product (DLP) was recorded for each patient. Effective dose was estimated by multiplying the DLP reported by the device by a conversion factor of 0.026 mSv/mGy∙cm [13].

### CT scanning protocol and image reconstruction

**Fig. 1:**
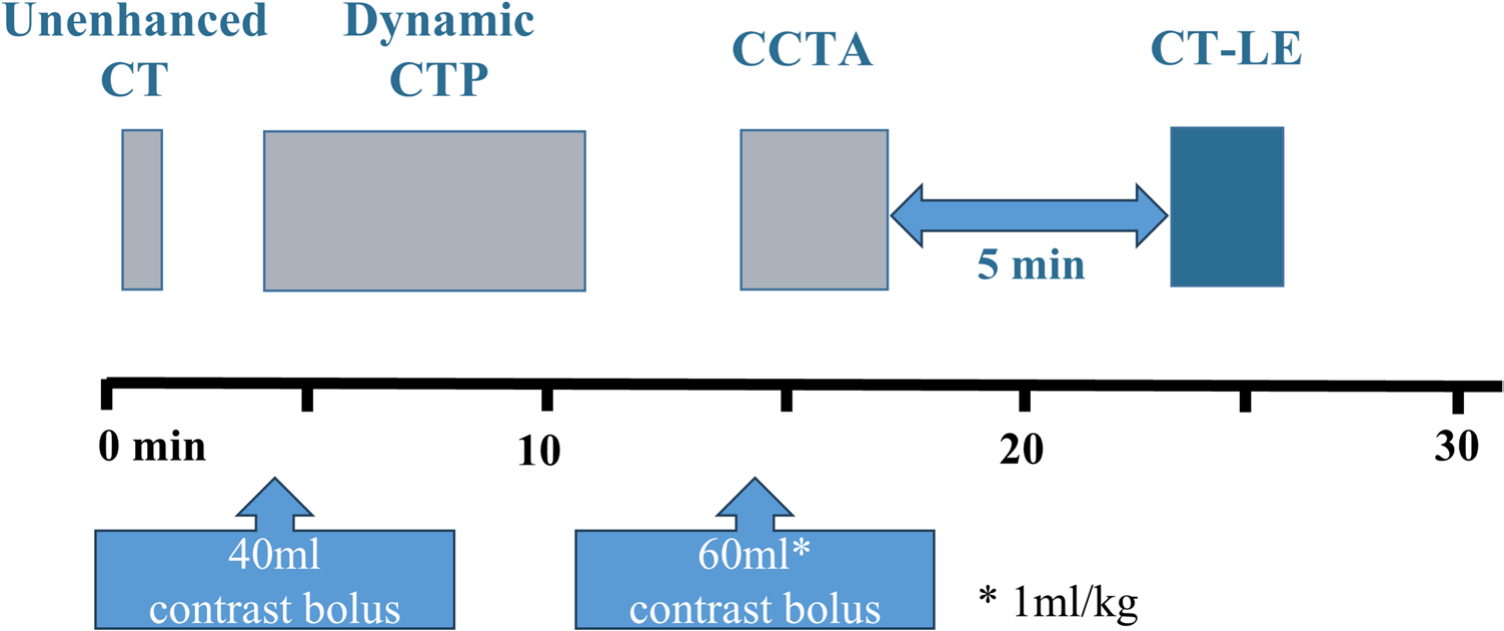
Cardiac CT protocol. *CTP* CT perfusion, *CCTA* coronary CT angiography, *CT-LE* CT late enhancement

All examinations were performed using a 320-row multidetector CT system (Aquilion ONE Nature edition; Canon Medical Systems, Otawara, Japan). The cardiac CT protocol consisted of unenhanced CT, stress dynamic CTP, CCTA, and CT-LE (Fig. 1). Dynamic CTP was performed with a bolus injection of 40 mL of iohexol (flow rate, 4 mL/s) with an iodine concentration of 300 mgI/mL (Iohexol 300; Fuji Pharma, Tokyo, Japan) followed by 20 mL of saline (flow rate, 4 mL/s). CCTA was performed with bolus injection of iohexol at 1 mL/kg (flow rate, 4 mL/s) followed by 20 mL of saline (flow rate, 4 mL/s). End-systolic (40% of the cardiac cycle) CT-LE was acquired using prospectively electrocardiogram-triggered axial scans at 5 min after CCTA [14, 15]. Tube potential was 80 kVp and tube current was 400 mA. Gantry rotation time was 0.275 s.

Images were reconstructed with a slice thickness of 1.0 mm with a 512 × 512 matrix combined with three different algorithms: (a) SR-DLR (Precise IQ Engine (PIQE); Canon Medical Systems), (b) C-DLR (Advanced intelligent Clear-IQ Engine (AiCE); Canon Medical Systems); and (c) hybrid IR (adaptive iterative dose reduction 3D (AIDR 3D), FC03, Canon Medical Systems). Details concerning the training of the DCNN for SR-DLR have been presented previously [11]. The settings for AIDR 3D, PIQE and AiCE have three selectable levels of reconstruction strength (Mild, Standard, or Strong) to control the amount of noise reduction. This study used the highest strength.

### Image analysis of CT-LE

#### Quantitative image analysis of CT-LE

Quantitative image analyses were conducted by an experienced radiologist (MT, 8 years of experience in cardiac CT) who was blinded to the clinical information and results of other diagnostic tests from subjects. To measure the CT number, an elliptical region of interest, 5 cm^2^ in the left ventricular cavity (LVC) and larger than 1 cm^2^ in the left ventricular septal myocardium (LVM) were placed on axial CT-LE datasets with a slice thickness of 5 mm [10]. If a myocardial scar was observed, a polygonal region of interest (ROI) as large as possible was also placed in that region. When multiple scars were observed in the same patient, the largest scar was used for the analysis. Image noise was measured as the standard deviation (SD) of the CT number within ROIs in the LVC and LVM. We also determined the signal-to-noise ratio (SNR) and contrast-to-noise ratio (CNR) on a patient-by-patient basis with the following equations:

$$\begin{gathered} {\mathbf{SNR}} \, = \, {\mathbf{HU}}/{\mathbf{noise}} \hfill \\ {\mathbf{CNR}} \, = \, \left[ {{\mathbf{HU}}\left( {{\mathbf{scar}}} \right){-}{\mathbf{HU}}\left( {{\mathbf{remote}}} \right)} \right]/{\mathbf{noise}}\left( {{\mathbf{remote}}} \right) \hfill \\ \end{gathered}$$

#### Qualitative image analysis of CT-LE

For qualitative image analysis, a total of 90 datasets (30 patients × 3 different image reconstructions) were presented in random order to two radiologists (M.T. and S.O.).Qualitative image quality was assessed using a 5-point Likert scale (5: excellent, 4: good, 3: moderate, 2: poor, 1: non-diagnostic) to evaluate each of the following parameters: noise reduction, sharpness, visibility of scar and myocardial boarder. Additionally, the overall image quality was evaluated. The mean scores from the two observers were reported as the final image quality scores.

#### Visual assessment of myocardial scar

Transmural extent of the infarct and non-ischemic fibrosis were evaluated by an experienced radiologist (MT, 8 years of experience in cardiac CT) and a less-experienced radiologist (S.O., with 2 years of experience in cardiac CT) at the AHA 17 segment level using CT-LE images reconstructed with three different methods. The transmural extent of the infarct was assessed on a 4-point scale (1–25%, 26–50%, 51–75%, and 76–100%). When a myocardial infarct or non-ischemic fibrosis was detected, observers were also instructed to manually trace and measure the largest short-axis area to evaluate the reproducibility of scar sizing [4].

### Statistical analysis

All continuous variables are expressed as median and IQR or mean ± SD, as appropriate. The Friedman test and the Wilcoxon matched-pairs signed-rank test were used to analyze differences in CT number, image noise, SNR, CNR, qualitative image quality scores, and inter-observer difference in scar sizing among SR-DLR, C-DLR and hybrid IR CT-LE images. The degree of inter-observer agreement of the transmural extent of the infarct and non-ischemic fibrosis at segment level was evaluated using weighted Cohen kappa coefficient. The degree of inter-observer agreement of the qualitative image quality score was evaluated using weighted Cohen kappa coefficient and adjacent agreement rate, which measures the proportion of ratings that either perfectly matched or differed by only one category [16]. The degree of inter-observer agreement of the scar sizing was evaluated using Bland–Altman method. Differences were considered statistically significant at the level of *p* < 0.05 with Bonferroni correction. All analyses were performed using SPSS version 28.0.1.1 software (International Business Machines, Inc., Armonk, New York, USA).

## Results

### Patient characteristics and radiation doses

Patient characteristics are shown in Table 1. Heart rate during CT-LE was 61 beats/min (IQR, 58–65 beats/min). DLP and estimated effective radiation dose for CT-LE were 42 mGy∙cm (IQR 36–54 mGy∙cm) and 1.1 mSv (IQR 0.9–1.4 mSv), respectively. The contrast dose for CT-LE was 98 mL (IQR 96–100 mL).

**Table 1:** Patient characteristics

Patient characteristics	*n* = 30
Sex	
Men, *n* (%)	19 (63)
Women, *n* (%)	11 (38)
Age (years), median (IQR)	73 (61–77)
Body mass index(kg/m^2^), median (IQR)	24.1 (22.0–27.0)
Risk factor, *n* (%)	
Hypertension	18 (60%)
Diabetes mellitus	5 (17%)
Hyperlipidemia	10 (33%)
Smoking	12 (40%)
Family history of CAD	7 (23%)
History of myocardial infarction, *n* (%)	5 (17%)
Prior-PCI	10 (33%)

*IQR* inter quantile range, *CAD* coronary artery disease, *PCI* percutaneous coronary intervention

### Prevalence of myocardial scars

**Fig. 2:**
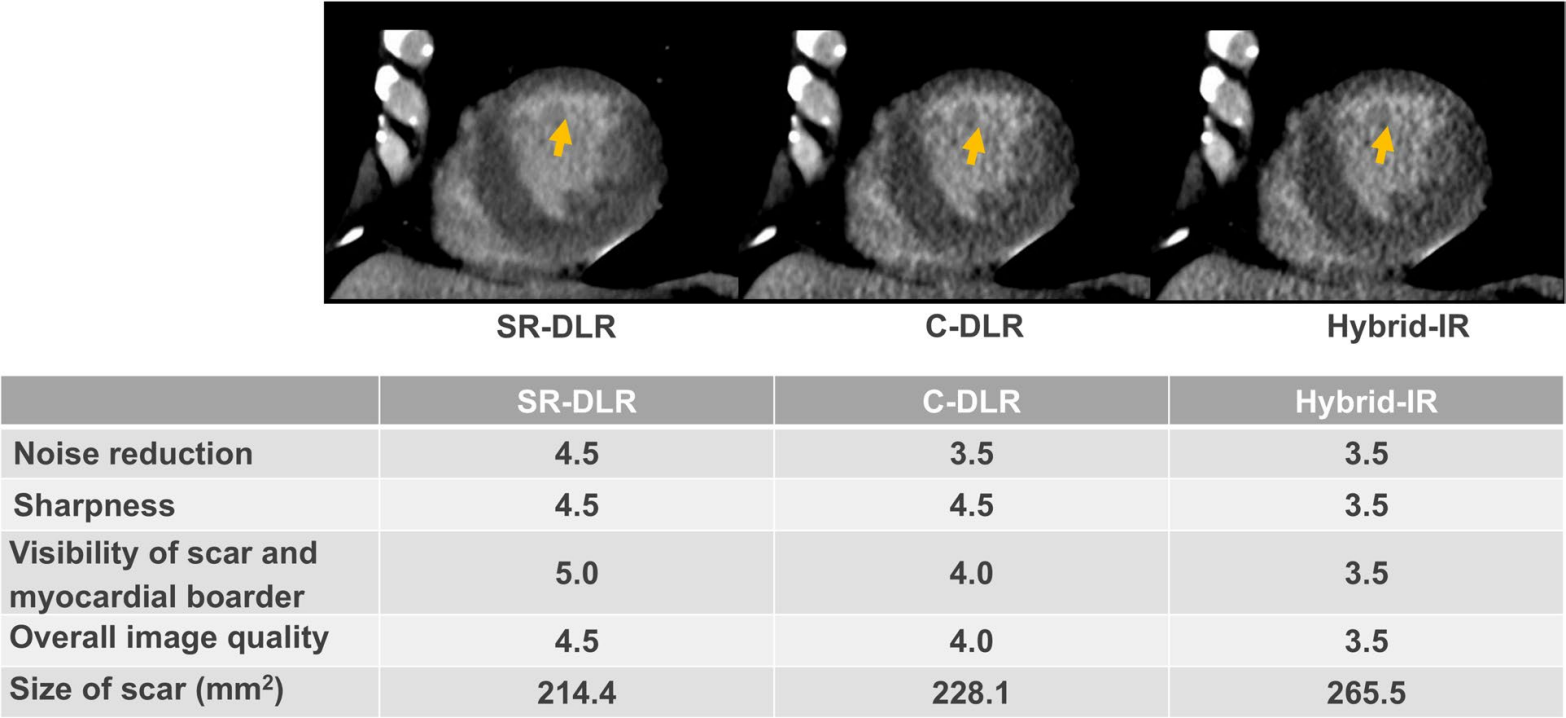
A 56-year-old man with smoking habits. Subendocardial infarction is observed in the anterior wall. Image noise is reduced with SR-DLR compared to C-DLR or hybrid IR. In the SR-DLR image, contrast between the infarct and lumen is more distinct at the border. *SR-DLR* super-resolution deep learning reconstruction, *C-DLR* conventional deep learning reconstruction, *hybrid IR* hybrid iterative reconstruction

**Fig. 3:**
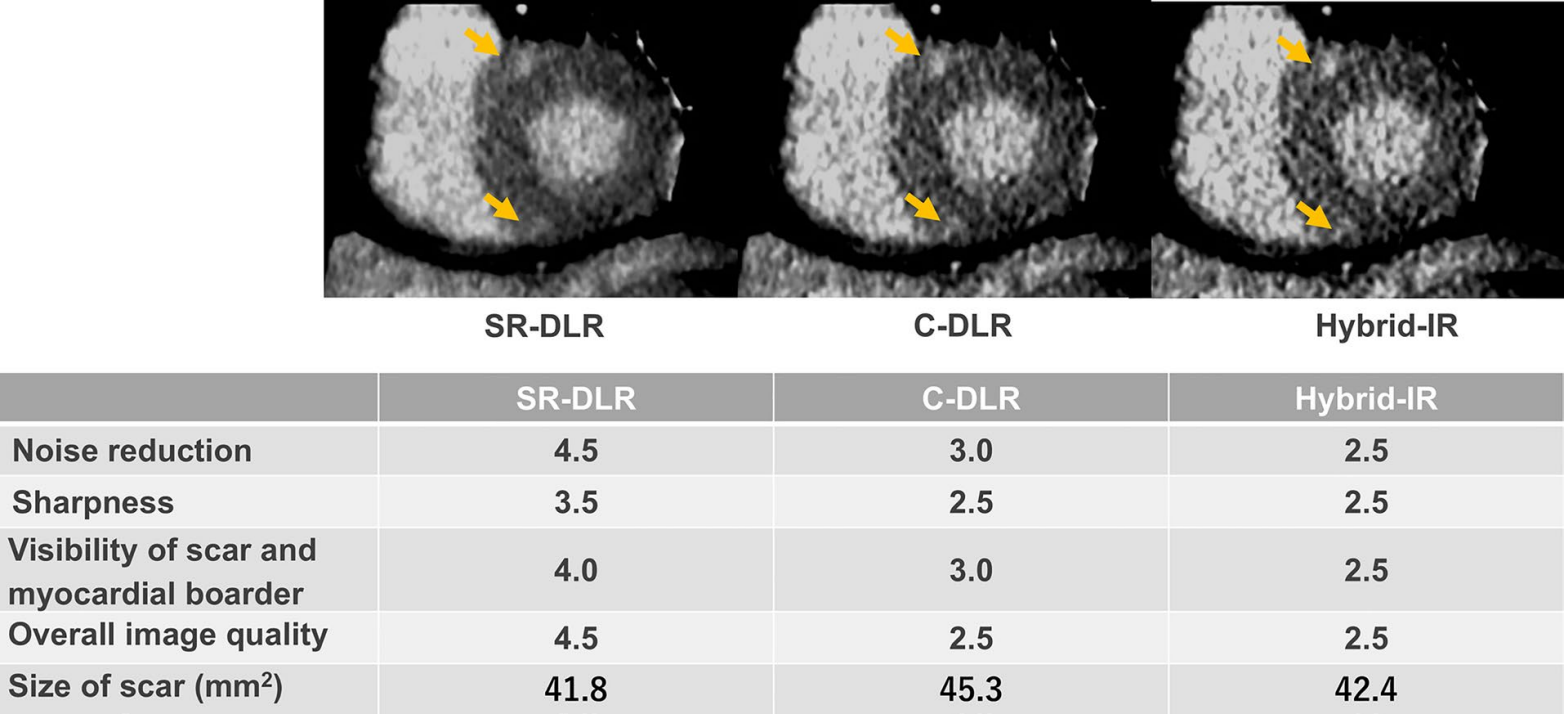
An 89-year-old woman with hypertension and dyslipidemia. Myocardial fibrosis in the midwall of the right ventricular insertion is more easily detected on SR-DLR images than on conventional DLR or hybrid IR. *SR-DLR* super-resolution deep learning reconstruction, *C-DLR* conventional deep learning reconstruction, *hybrid IR* hybrid iterative reconstruction

Myocardial scar was observed on CT-LE in 10 patients (33%, 10/30). All five patients with a history of myocardial infarction exhibited myocardial scar with an ischemic pattern in the regions where percutaneous coronary intervention (PCI) was performed. Among the remaining five patients, myocardial scar was observed with an ischemic pattern in three and a non-ischemic pattern in two. Of those without a history of myocardial infarction but showing an ischemic pattern (Fig. 2), two had a history of undergoing PCI, and one had significant coronary artery stenosis on CCTA. Both patients with a non-ischemic pattern demonstrated non-specific late enhancement at the RV insertion (Fig. 3), but neither was definitively diagnosed with non-ischemic cardiomyopathy. Myocardial scars were detected in 54 (10.6%), 53 (10.4%), and 49 (9.6%) segments on SR-DLR, C-DLR, and hybrid IR, respectively. The prevalence of myocardial scar in CT-LE reconstructed with SR-DLR was significantly higher than those with hybrid IR (*p* = 0.02), while there was no significant difference between SR-DLR and C-DLR, and between C-DLR and hybrid IR (*p* > 0.05).

### Image quality of CT-LE

Image quality of CT-LE using SR-DLR, C-DLR and hybrid IR is shown in Table 2.

**Table 2:** Image quality of CCTA using SR-DLR and C-DLR

	SR-DLR	C-DLR	Hybrid-IR	*p* value*	*p* value^†^		
					SR-DLR vs C-DLR	SR-DLR vs Hybrid-IR	C-DLR vs Hybrid-IR
Quantitative image quality							
LV cavity, median (IQR)							
HU	106.0(96.1–122.3)	104.3(93.3–121.9)	103.7(93.5–121.1)	< 0.001	< 0.001	< 0.001	1.00
Image noise (HU)	6.2(5.6–7.2)	9.6(8.4–10.7)	9.8(8.5–12.0)	< 0.001	< 0.001	< 0.001	0.21
SNR	17.6(13.9–21.0)	11.3(9.4–13.4)	11.3(8.6–13.6)	< 0.001	< 0.001	< 0.001	0.36
Myocardium, median (IQR)							
HU	77.0(70.2–87.4)	76.2(67.1–85.8)	76.6(68.0–85.6)	< 0.001	< 0.001	< 0.001	1.00
Image noise (HU)	6.4(5.2–7.1)	9.4(8.0–10.1)	9.6(8.1–11.3)	< 0.001	< 0.001	< 0.001	0.36
SNR	13.2(11.2–14.7)	8.6(7.5–9.3)	8.6(7.1–9.2)	< 0.001	< 0.001	< 0.001	0.36
Scar							
CNR	4.7(4.2–4.9)	3.0(2.8–3.6)	3.3(2.9–3.5)	< 0.001	< 0.001	0.04	0.22
Size of scar	211.9(153.8–356.5)	207.2(149.0–408.1)	217.1(148.5–446.3)	0.12	1.00	0.13	0.54
Inter-observer difference in scar sizing	62.7(22.1–144.4)	103.8(58.2–170.5)	130.6(82.2–195.6)	< 0.01	1.00	0.02	0.02
Qualitative image quality							
Noise reduction	4.5(4.0–4.5)	3.5(3.0–3.5)	3.0(2.5–3.5)	< 0.001	< 0.001	< 0.001	0.09
Sharpness	4.0(3.5–4.5)	3.5(3.0–3.5)	3.0(2.5–3.5)	< 0.001	< 0.001	< 0.001	0.06
Visibility of scar and myocardial boarder	4.0(3.5–4.5)	3.5(3.0–4.0)	3.5(2.5–3.5)	< 0.001	< 0.001	< 0.001	0.09
Overall image quality	4.0(3.9–4.5)	3.5(3.0–3.5)	3.0(2.5–3.5)	< 0.001	< 0.001	< 0.001	0.04

*SR-DLR* super-resolution deep learning reconstruction, *C-DLR* conventional deep learning reconstruction, *hybrid IR* hybrid iterative reconstruction, *IQR* inter quantile range, *HU* Hounsfield unit, *SNR* signal-to-noise ratio, *CNR* contrast-to-noise ratio

^*^Friedman test

^†^Wilcoxon matched-pairs signed-rank test

#### Quantitative image quality

**Fig. 4:**
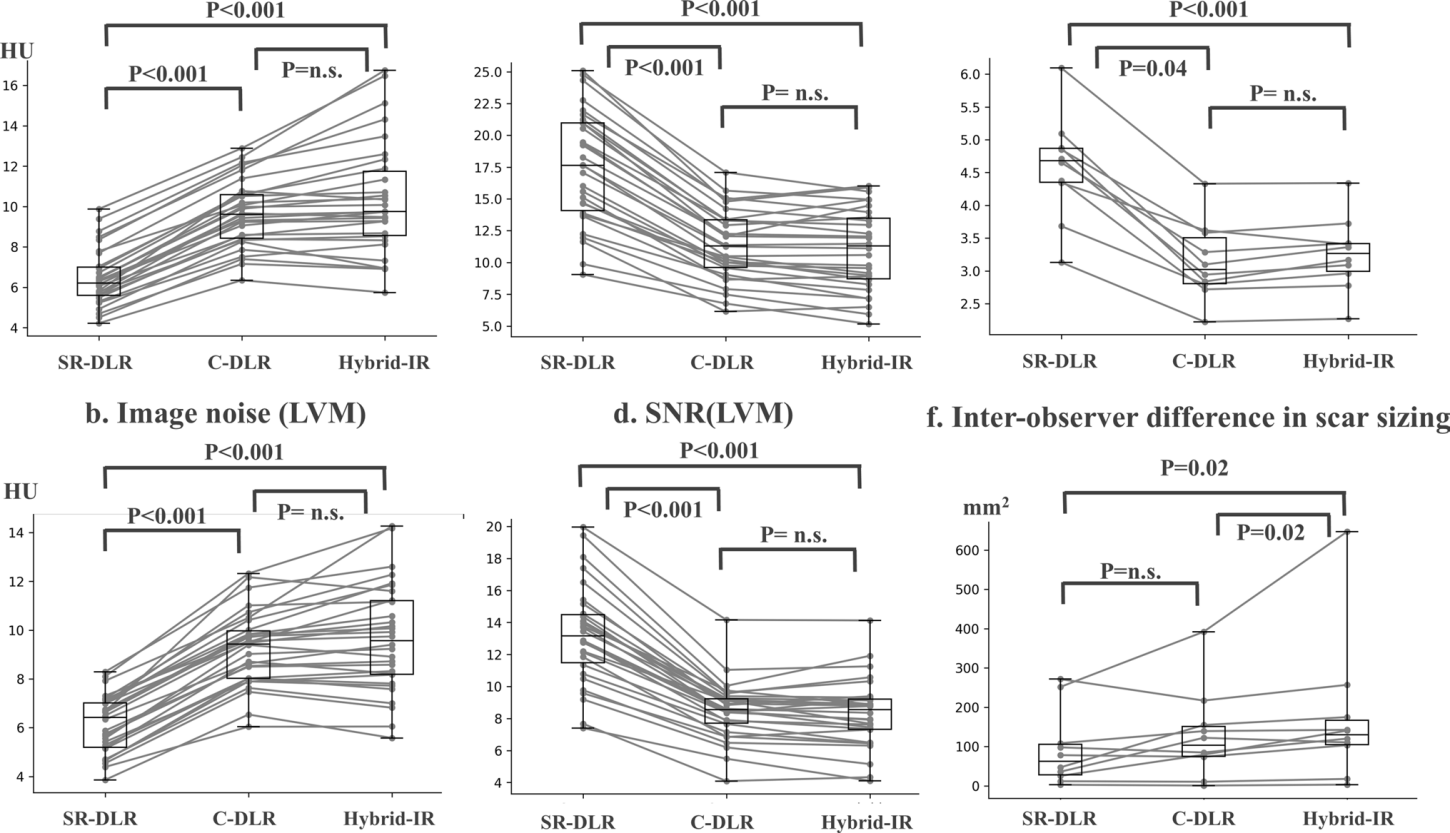
Comparison of image noise in LVC (**a**) and LVM (**b**), SNR of LVC (**c**) and LVM (**d**), CNR (**e**) and inter-observer difference in scar sizing (**f**) among SR-DLR, C-DLR and hybrid IR images. Center lines represent median values, with box limits indicating 25th and 75th percentiles, and whiskers extending from minimum to maximum values. All p-values were calculated using the Wilcoxon matched-pairs signed-rank test. *SR-DLR* super-resolution deep learning reconstruction, *C-DLR* conventional deep learning reconstruction, *hybrid IR* hybrid iterative reconstruction, *LVC* left ventricular cavity, *LVM* left ventricular myocardium, *HU* Hounsfield units, *SNR* signal-to-noise ratio, *CNR* contrast-to-noise ratio

SR-DLR significantly reduced image noise in the LVC and LVM by 35% and 32% respectively, compared to C-DLR (LVC: median 6.2 HU, IQR 5.6–7.2 HU vs 9.6 HU, IQR 8.4–10.7 HU, *p* < 0.001; LVM: 6.4 HU, IQR 5.2–7.1 HU vs 9.4 HU, IQR 8.0–10.1 HU, *p* < 0.001) and by 37% and 33% respectively, compared to hybrid IR (LVC: 9.8 HU, IQR 8.5–12.0 HU, *p* < 0.001; LVM, 9.6 HU, IQR 8.1–11.3 HU, *p* < 0.001). The SR-DLR method significantly improved SNR in LVC and LVM compared with C-DLR (LVC: 17.6, IQR 13.9–21.0 vs 11.3, IQR 9.4–13.4, *p* < 0.001; LVM: 13.2, IQR 11.2–14.7 vs 8.6, IQR 7.5–9.3; *p* < 0.001), and hybrid IR (LVC: 11.3, IQR 8.6–13.6, *p* < 0.001; LVM: 8.6, IQR 7.1–9.2, *p* < 0.001). Likewise, CNR was significantly improved compared with C-DLR (4.7, IQR 4.2–4.9 vs 3.0, IQR 2.8–3.6, *p* < 0.001) and hybrid IR (3.3, IQR 2.9–3.5, *p* = 0.04) (Fig. 4).

#### Qualitative image quality

Qualitative image quality of CT-LE using SR-DLR, C-DLR and hybrid IR is shown in Table 2. The weighted Cohen kappa coefficient for image quality scores was 0.23 for noise reduction, 0.13 for sharpness, 0.13 for visibility of scar and myocardial boarder, and 0.17 for overall image quality. The adjacent agreement rates were 86.7% for noise reduction, 76.7% for sharpness, 78.9% for visibility of scar and myocardial border, and 84.4% for overall image quality (Table 3). SR-DLR achieved the highest image quality scores in terms of noise reduction, sharpness, visibility of scar and myocardial boarder, and overall image quality compared to C-DLR and hybrid IR (all *p* < 0.001). The overall image quality was significantly higher for CT-LE reconstructed using C-DLR than for hybrid IR (*p* = 0.04), while differences in other parameters between C-DLR and hybrid IR were not statistically significant.

**Table 3:** Inter-observer agreement of qualitative image quality

Observer 2	Observer 1				
	1	2	3	4	5
A: Noise reduction					
1	0	0	0	0	0
2	1	3	9	7	1
3	0	1	15	26	4
4	0	0	0	3	18
5	0	0	0	0	2
B: sharpness					
1	0	2	0	0	0
2	1	3	6	9	0
3	0	2	10	28	12
4	0	0	2	5	10
5	0	0	0	0	0
C: visibility of scar and myocardial border					
1	0	1	0	0	0
2	0	3	7	7	1
3	0	2	6	29	11
4	0	0	3	10	7
5	0	0	0	1	2
D: overall image quality					
1	0	1	0	0	0
2	1	4	7	5	0
3	0	1	9	31	9
4	0	0	2	7	13
5	0	0	0	0	0

#### Visual assessment of myocardial scar

The inter-observer agreement for myocardial scar grading was slightly improved with SR-DLR compared to C-DLR and hybrid IR (weighted kappa: SR-DLR, 0.66; C-DLR, 0.63; hybrid IR, 0.63).

**Fig. 5:**
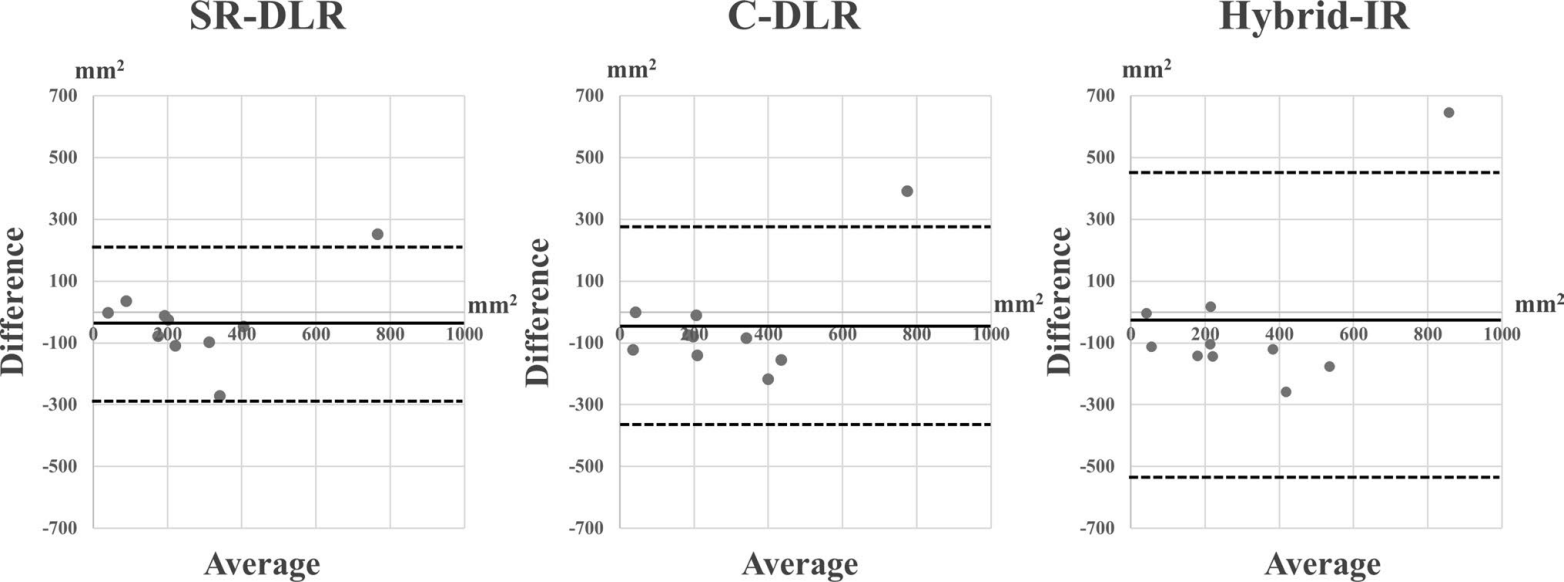
Bland–Altman plots with 95% limits of agreement demonstrate agreement between two observers in myocardial scar sizing. *SR-DLR* super-resolution deep learning reconstruction, *C-DLR* conventional deep learning reconstruction, *hybrid IR* hybrid iterative reconstruction

Inter-observer difference in scar sizing is reduced with SR-DLR (62.7 mm^2^, IQR 22.1–144.4 mm^2^) and C-DLR (103.8 mm^2^, IQR 58.2–170.5 mm^2^) compared with hybrid IR (130.6 mm^2^, IQR 82.2–195.6 mm^2^) (both *p* = 0.02) (Fig. 4f). There was no significant difference in the size of scar (*p* = 0.12). Bland–Altman plots showed a mean difference between observers of −35.7 ± 131.8 mm^2^ (95% limits of agreement [LoA], −294.0 to 222.7 mm^2^) for SR-DLR, −49.2 ± 168.1 mm^2^ (95% LoA, −378.7 to 280.3 mm^2^) for C-DLR, and −38.9 ± 253.4 mm^2^ (95% LoA, −535.6 to 457.8 mm^2^) for hybrid IR (Fig. 5).

## Discussion

This study evaluated the impact of SR-DLR in CT-LE in vivo, yielding the following findings: (1) SR-DLR substantially reduced image noise by more than 30%, compared to C-DLR and hybrid IR; (2) image quality was significantly improved with SR-DLR compared to C-DLR or hybrid IR; (3) inter-observer agreement in scar sizing was significantly improved with SR-DLR and C-DLR compared with hybrid IR.

CT-LE involves injection of a contrast medium to evaluate delayed enhancement of the myocardium [17]. In optimizing the imaging parameters, increasing myocardial contrast enhancement is essential as the contrast between remote and hyper-enhanced myocardium is much lower in CT-LE than in LGE-MRI [18]. Low tube potential imaging represents an effective approach for CT-LE, permitting a reduction in radiation dose and augmented contrast enhancement. However, this approach also carries the disadvantage of increasing noise.

The iterative reconstruction method has been developed to reduce image noise in CT, which is commonly associated with the use of standard filtered back projection (FBP) reconstruction. Subsequent studies have demonstrated the efficacy of hybrid IR in enhancing image quality from cardiac CT examinations in clinical settings [8]. A previous study demonstrated that hybrid IR decreased image noise in CT-LE by 30% compared with FBP [8]. In recent years, image reconstruction algorithms using DCNN have been applied to cardiac CT [10, 19]. Tatsugami et al. [19] demonstrated that C-DLR decreased image noise of CCTA by 20% compared with hybrid IR, while Takafuji et al. found that C-DLR decreased image noise of dynamic CTP by 20% compared with hybrid IR. In the present study, C-DLR slightly reduced the image noise for CT-LE compared with hybrid IR. The relatively small difference in image noise between CT-LE by C-DLR and hybrid IR may be attributable to the lower image noise of CT-LE by hybrid IR compared to that in CCTA or dynamic CTP in previous studies [10, 20]. The present study evaluated CT-LE images with a thickness of 5 mm, and used a higher tube current for CT-LE than that used for dynamic CTP in a previous study [10], contributing to the lower noise observed in hybrid IR images in our study than in the aforementioned previous studies.

More recently, SR-DLR trained using data acquired from UHR-CT has become available to CCTA [12, 19, 21]. In addition to enhancing spatial resolution, the SR-DLR also exhibits effective noise reduction. Previous studies [12, 19, 21] have shown that SR-DLR decreased image noise of CCTA by 14–31% compared to C-DLR and by 32 to 35% compared to hybrid IR. In the present study, SR-DLR decreased image noise of CT-LE by more than 30% and improved the image quality of CT-LE compared to C-DLR and hybrid IR. The mean DLP in CT-LE has been reported to range from 36 to 165 mGy∙cm [4, 8, 15, 22]. In the present study, the median DLP and the effective radiation dose for CT-LE were 42 mGy∙cm and 1.1 mSv, respectively, using a tube potential of 80 kV and prospective electrocardiography-triggered scans. As image noise correlates inversely with the square root of the tube current [23], 37% noise reduction observed in the LVC with the SR-DLR technique, compared to hybrid IR, theoretically enables a reduction in tube current and consequently, radiation exposure by approximately 60% in CT-LE, while maintaining the same image noise level. Further investigations are warranted to assess the image quality and diagnostic accuracy for myocardial scar assessment when CT-LE is performed at low tube current and reconstructed using SR-DLR.

Our finding that the CT numbers of LVC and LVM in CT-LE reconstructed by SR-DLR were slightly but significantly higher than those reconstructed by C-DLR and hybrid IR was in line with previous phantom study [24]. This slight difference in CT value may potentially influence the quantification of extracellular volume fraction by CT-LE. Further validation of the ECV measurements by CT-LE reconstructed with SR-DLR is necessary.

In the current study, inter-observer agreement for the transmural extent of the infarct and non-ischemic fibrosis was slightly improved with SR-DLR compared to C-DLR and hybrid IR. Furthermore, SR-DLR significantly reduced inter-observer differences in myocardial scar sizing compared to hybrid IR. The combination of reduced image noise and improved sharpness by SR-DLR enhances the visualization of myocardial scars. The reproducible assessment of myocardial scar extent using CT-LE reconstructed with SR-DLR may support clinical decision-making in patients with cardiac disease.

There are several limitations to this study. First, this was a single-center study with a small number of patients. More data derived from larger populations in a multi-center setting are needed to confirm our initial observations. Second, while three levels of reconstruction strength (Mild, Standard, Strong) are selectable to control the amount of noise reduction for SR-DLR, C-DLR, and hybrid IR, this study only evaluated a single strength level (Strong). Further evaluations are warranted to determine the optimal intensity level for the DLR algorithm and the degree of reduction in radiation exposure that can be achieved using DLR. Finally, validation against LGE-MRI could not be performed as none of the patients in this study underwent LGE-MRI. A further validation study is required to assess the concordance between myocardial scars identified by CT-LE using DLR and those identified by LGE-MRI.

In conclusion, SR-DLR improved image noise and image quality for myocardial CT-LE compared with C-DLR and hybrid IR techniques, and improved inter-observer reproducibility of scar sizing compared to hybrid IR. The SR-DLR approach holds promise for improving the assessment of myocardial scarring by CT-LE.

## Abbreviations

CAD: Coronary artery disease
CCTA: Coronary computed tomography angiography
C-DLR: Conventional deep learning image reconstruction
CT: Computed tomography
CT-LE: Computed tomography late enhancement
CTP: Computed tomography perfusion
DCNN: Deep convolutional neural network
HU: Hounsfield units
IR: Iterative reconstruction
LGE: Late gadolinium enhancement
LVC: Left ventricular cavity
LVM: Left ventricular myocardium
MRI: Magnetic resonance imaging
ROI: Region of interest
SD: Standard deviation
SR-DLR: Super-resolution deep learning image reconstruction
UHR-CT: Ultra-high-resolution computed tomography
